# The Influence of Physical Activity during Pregnancy on Maternal Pain and Discomfort: A Meta-Analysis

**DOI:** 10.3390/jpm14010044

**Published:** 2023-12-28

**Authors:** Miguel Sánchez-Polán, Taniya S. Nagpal, Dingfeng Zhang, Cristina Silva-Jose, Rocío Montejo, Rubén Barakat

**Affiliations:** 1 AFIPE Research Group, Faculty of Physical Activity and Sport Sciences-INEF, Universidad Politécnica de Madrid, 28040 Madrid, Spain; miguel.sanchez.polan@upm.es (M.S.-P.); dingfeng.zhang@alumnos.upm.es (D.Z.); cristina.silva.jose@upm.es (C.S.-J.); 2 Faculty of Kinesiology, Sport, and Recreation, University of Alberta, Edmonton, AB T6G 2R3, Canada; tnagpal@ualberta.ca; 3 Department of Obstetrics and Gynecology, Institute of Clinical Sciences, Sahlgrenska Academy, University of Gothenburg, 405 30 Gothenburg, Sweden; rocio.montejo.rodriguez@gu.se; 4 Department of Obstetrics and Gynecology, Sahlgrenska University Hospital, 413 46 Gothenburg, Sweden

**Keywords:** maternal pain, low back pain, pelvic girdle pain, leg pain, labour pain, pain intensity, physical activity, pregnancy

## Abstract

Pregnant women may experience pain and discomfort during pregnancy, especially in areas such as the lower back and pelvic girdle. Pain in pregnancy is associated with poor quality of life, and because it is a common occurrence, pregnant women may be offered several resources to prevent discomforts throughout pregnancy, such as engaging in physical activity. This study was a meta-analysis of randomised controlled trials (prospectively registered in Prospero, registration number: CRD42023451320) aimed to assess the effects of physical activity during pregnancy on maternal pain and discomfort. We analysed 16 randomised clinical trials. The results of these analyses indicate that women who performed physical activity had significantly less intensity of pain (z = <2.69, *p* = <0.007; SMD = −0.66, 95% CI = −1.13, −0.18, I^2^ = <91%, P_heterogeneity_ = <0.001) and a reduction observed in the disability questionnaire (z = <2.37, *p* = <0.02; SMD = −0.80, 95% CI = −1.47, −0.14, I^2^ = <91%, P_heterogeneity_ = <0.001), and overall reduced general pain (z = <3.87, *p* = <0.001; SMD = −0.56, 95% CI = −0.84, −0.27, I^2^ = <86%, P_heterogeneity_ = <0.001) than women who did not practice physical activity during pregnancy. In conclusion, physical activity during pregnancy could effectively help to diminish pain intensity, reduce disability due to pain, and generally reduce pain.

## 1. Introduction

Pregnancy increases the risk of musculoskeletal discomfort and pain due to physiological demands placed on the body, such as an increase in weight gain, abdominal pressure, hormonal fluctuations, and fluid retention. Pelvic girdle pain (PGP) and low back pain (LBP) emerge as prevalent complications, and may also have a negative impact on quality of life among expectant mothers [1]. Although the pathophysiology is not fully understood and consists of multiple potential pathways [2,3,4], PGP and LBP during pregnancy can be attributed to various factors, with one of the most relevant being the increase in uterus weight and the subsequent expansion of the abdominal space. The various discomforts experienced by pregnant women can intensify over the course of pregnancy and can impact other aspects of their well-being, such as sleep quality.

Positively, several options are available to support pregnant women with both the prevention and management of physical discomforts, including counselling to adopt appropriate spinal positions, pillows to support sleep, belts for assisting with weight displacement, and engagement in physical activities such as pelvic floor muscle training and resistance exercise [1,5,6,7]. In particular, physical activity has emerged as one of the most commonly suggested preventive therapies for pain during pregnancy, although its efficacy is not yet fully acknowledged. Various forms of physical activity during pregnancy have demonstrated a high level of effectiveness in improving pain-related outcomes, such as both aerobic exercise and swimming, without posing significant risks in terms of negative prenatal, perinatal, and postnatal outcomes [8]. In fact, a systematic review and meta-analysis that included 32 studies found that pregnant women who were active during gestation reported lower pain during pregnancy and early postpartum compared to those who were not active [9]. 

The scientific literature on the effectiveness of prenatal physical activity and musculoskeletal pain and discomfort is inconsistent, with some studies suggesting positive results, while others show null effects. For example, in the same review mentioned above, although the severity of pain decreased when women were active, physical activity did not reduce the odds of low back pain, pelvic girdle pain, or lumbopelvic pain [9]. Similar findings were shown in a recent review that examined randomised controlled trials on exercise during pregnancy and the effect on low back pain and pelvic girdle pain, with findings demonstrating improvement in the ability of the active population to manage pain, but without effect on the odds of having pain [10]. Another review specifically sought to summarise the literature on Pilates as a mode of exercise to mitigate pain during pregnancy, and suggested that it was effective in reducing low back pain, but the results were limited to those of only two studies [11]. 

The evidence base alludes to the effectiveness of physical activity during pregnancy, especially to manage the severity of pain; however, it is not clear whether or not physical activity can be an effective preventive modality. Previous reviews were primarily limited to a single language, were specific to pain regions, and also included low-to-moderate-quality evidence inclusive of observational studies. To go deeper into findings on the impact of prenatal exercise on musculoskeletal pain and discomfort, this systematic review and meta-analysis sought to examine randomised controlled trials on a variety of pain and discomfort indices and regions.

## 2. Materials and Methods

This article is a meta-analysis performed following the Preferred Reporting Items for Systematic Review and Meta-Analyses (PRISMA) assessment [12]. The protocol was previously registered in PROSPERO (Registration No.: CRD42023451320), and the review was guided by the PICOS (Participants, Interventions, Comparisons and Outcomes) framework. 

### 2.1. Population

Healthy pregnant women without contraindications to exercise during gestation [13,14], over 18 years old, who were enrolled in a regular physical activity programme during pregnancy were selected. 

### 2.2. Intervention

Intervention programmes based on physical activities during pregnancy were searched, retrieving the following characteristics: session time in minutes, intensity of physical activity, weekly frequency of the sessions, duration of intervention programme in weeks, type of exercise, supervision or not of the programme, and if reported, adherence of the participants to the intervention. 

### 2.3. Comparison

Women who received usual or standard care (that is, did not perform physical activity during pregnancy, belonging to the control group) were selected to compare the results with participants performing the intervention programme. Control groups were defined by each individual study and consisted of no physical activity intervention; in some cases, participants were given alternate information about generally having a healthy pregnancy, but they were not given the intervention itself. 

### 2.4. Outcomes

Primary outcomes were back, labour, legs, lumbopelvic and pelvic girdle pain, pain intensity and disability index, reported as the number of events presented in each group or measured as a result of a quantifiable and validated questionnaire including: the Numeric Pain Intensity Scale (NPIS), the Visual Analogue Scale (VAS), and the Oswestry Disability Index (ODI). 

### 2.5. Study Selection Process, Data Sources, and Data Extraction 

Randomised clinical trials, published after 2010 in English or Spanish, were assessed for eligibility. Systematic reviews that were published retrieving similar outcomes were searched.

Extensive searches were performed from July to September 2023, assessing results from the following electronic databases: Academic Search Premier, Education Resources Information Center (ERIC), Medline, SportDiscus, OpenDissertations (all of them through EBSCO platform), Clinicaltrials.gov, Web of Science (WoS), Scopus, Physiotherapy Evidence Database (PEDro), and Cochrane Database of Systematic Reviews. Search terms (in English and Spanish) were as follows:

- English: physical activity OR exercise OR fitness OR physical exercise OR sport OR walking OR cycling OR physical intervention AND pregnancy OR pregnant OR prenatal OR maternal OR antenatal OR perinatal AND randomized clinical trial OR randomized controlled trial OR RCT AND pain OR pain management OR pain relief OR pain control OR pain reduction OR discomfort OR low back pain OR lumbar pain OR lumbar spine pain OR nonspecific low back pain OR chronic low back pain OR pelvic girdle pain OR PGP OR pelvic pain OR pelvic dysfunction.
- Spanish: actividad física O ejercicio O fitness O ejercicio físico O deporte O caminar O bicicleta O intervención Y embarazo O embarazada O prenatal O maternal O antenatal O perinatal Y ensayo clínico aleatorizado O ensayo controlado aleatorizado O ECA Y dolor O gestion del dolor O Alivio del dolor O control del dolor O reducción del dolor O malestar O lumbalgia O dolor lumbar O dolor de columna O dolor lumbar no específico O lumbalgia crónica O dolor de cintura pélvica O dolor pélvico O disfunción pélvica.

Comprehensive searches were performed by two researchers, initially retrieving titles and abstracts for determined databases, and later, assessing article eligibility. After this first screening, two reviewers examined the full texts to determine whether the articles met the inclusion criteria. If a researcher thought that one study did not meet the criteria to be included, it was mandatory to reach a consensus between reviewers to ensure that the appropriate decision was made. If this consensus was not possible, a third researcher was consulted to make the final decision.

A reviewer performed the initial data extraction after the screening, onto an Excel sheet, and later, other researchers independently assessed the extracted information to ensure the validity of the data extraction and develop the analyses. Data that were extracted (in addition to main outcome data for the meta-analyses) and descriptively reported were reference ID (author last name and year of publication), country in which the article was developed, sample size (total and per study group), intervention characteristics, primary and secondary outcomes included in the study, and any cointervention.

### 2.6. Risk of Bias and Quality of Evidence 

Selected articles were evaluated through the Cochrane Handbook to assess potential risk of bias through the following sources: selection (how the randomisation process was performed and if it was appropriate for the design), performance (how the exposure of different factors affected the blinding of the study group participants), detection (the blinding outcome assessment per study group), attrition (large lost-to-follow-up rates or incomplete outcome data), and reporting (selective reporting of outcomes) bias, with each source of bias evaluated and varying from low, unclear, and high risk of bias [15]. 

Quality of evidence was assessed with the Grading of Recommendations Assessment, Development and Evaluation (GRADE) assessment, through the GRADEpro tool available online [16,17]. This tool allows the researcher to know the quality of evidence of each outcome by evaluating if there is severe risk of bias or not, and assessing the characteristics of the intervention, outcome, and the results of the performed analyses.

### 2.7. Statistical Analysis

Analyses were carried out with RevMan (Review Manager, in its 5.4 version). In the case that outcomes were reported as a continuous variable (i.e., scores of back and leg pain, pain intensity, and Oswestry Disability Index), standardised mean differences (SMD) were calculated through inverse variance (IV) analysis. If the result was reported as a categorical variable (i.e., current events of back pain and pelvic girdle pain at the end of pregnancy per group), the risk ratio (RR) was calculated through Mantel–Haenszel (M-H) analysis.

For both analyses, the average was determined with a weight system that evaluated the sample size per study group and the analysis. Random effects model was used and the statistical alpha power was set at 0.05. The variability of the articles included per analysis was assessed with the I^2^ statistic, taking into account the following ranges: low heterogeneity (25%), moderate heterogeneity (25–75%), and high heterogeneity (>75%). High heterogeneity was reported in two subgroup analyses of continuous variables; however, it was opted to not divide the articles into subgroups due to the low number of articles analysed in this review, believing that this approach could provide a better view of the study.

To evaluate the potential publication bias of both analyses performed, the Egger regression test was used. This assessment usually provides a significant bias when the *p* value < 0.1 [18].

## 3. Results

**Figure 1:**
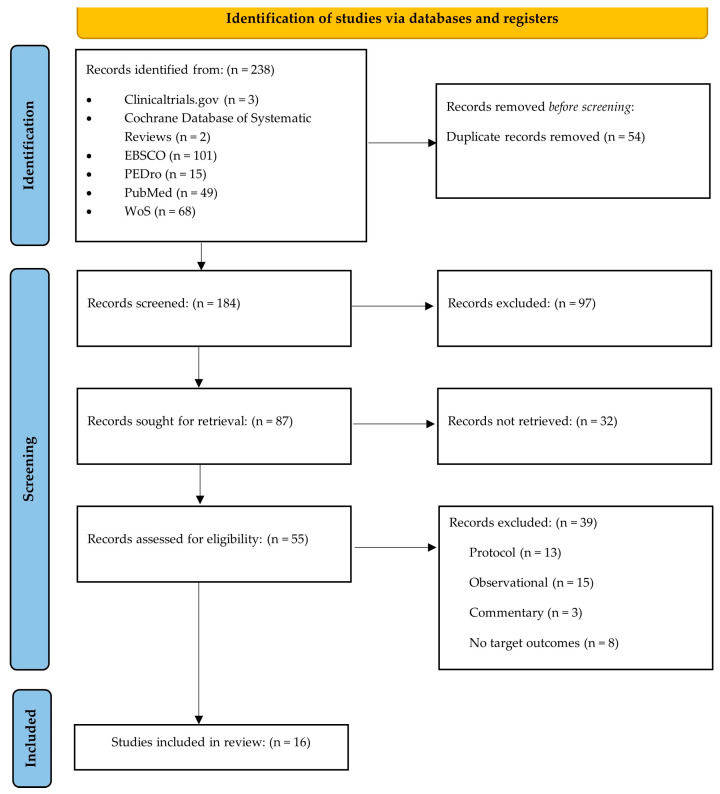
Flow diagram of the articles searched and analysed [19,20,21,22,23,24,25,26,27,28,29,30,31,32,33,34].

After searches, 238 articles were identified from selected electronic databases, initially excluding 54 of them due to deduplication. During the selection, 97 reports were excluded due to not meeting the inclusion criteria and 32 records were excluded due to their study design (non-randomised articles), and then 55 articles were screened in their full text. Finally, sixteen randomised clinical trials [19,20,21,22,23,24,25,26,27,28,29,30,31,32,33,34] were included, with the search process retrieved shown in Figure 1. 

### 3.1. Risk of Bias and Quality of Evidence Assessments

**Figure 2:**
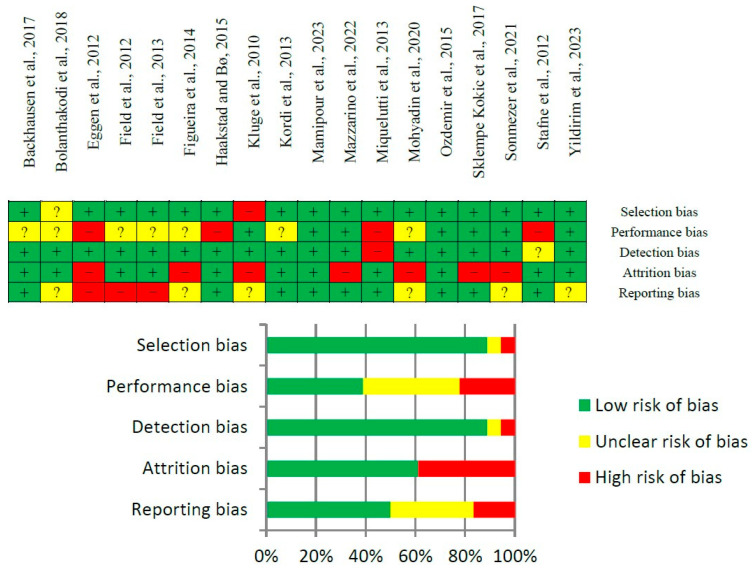
Risk of bias evaluation of analysed articles [19,20,21,22,23,24,25,26,27,28,29,30,31,32,33,34].

The quality of the evidence was high overall, due to the characteristics of the intervention and the absence of severe risk of bias. Generally, the risk of bias in the retrieved articles was low. However, the performance source of bias presented higher rates of unclear than low risk of bias, and we also had large rates of high risk and unclear risk of bias in attrition and reporting source of bias, respectively. However, it was opted to not exclude any article because the majority of the risk of bias was low and the quality of evidence was high (Figure 2).

### 3.2. Article Characteristics

Eight countries were included and reported in the 16 randomised clinical trials analysed, involving a total of 2613 participants. The articles included low-to-moderate-intensity physical activity programmes, lasting from 4 to 22 weeks during pregnancy, with sessions ranging from 20 to 70 min in duration. Of the 16 articles, 12 were supervised physical activity classes included in their intervention programme. Sociodemographic, intervention, and outcome characteristics are reported in Table 1.

### 3.3. Effect of Physical Activity on Pain, Pain Intensity, and ODI Score 

**Figure 3:**
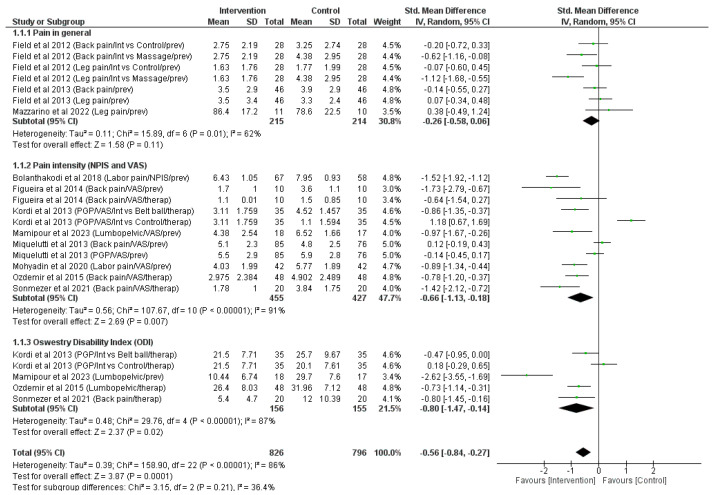
Meta-analysis of the effects of physical activity interventions on pain, pain intensity, and ODI score of retrieved articles [20,22,23,24,27,28,29,30,31,33]. Int (intervention); Prev (preventative effect of intervention); Numeric Pain Intensity Scale (NPIS); Visual Analogue Scale (VAS); Therap (therapeutic effect of intervention); PGP (pelvic girdle pain).

Eleven articles [20,22,23,24,27,28,29,30,31,33] (*n* = 931) were evaluated with respect to pain, intensity of pain and ODI questionnaire score. With regards to pain, there were no significant differences observed between groups (z = <1.58, *p* = <0.11; SMD = −0.26, 95% CI = −0.58, 0.06, I^2^ = <62%, P_heterogeneity_ = <0.01). However, a significant decrease was reported in participants in the intervention group at the end of the pregnancy for pain intensity, compared to women in the control group (z = <2.69, *p* = <0.007; SMD = −0.66, 95% CI = −1.13, −0.18, I^2^ = <91%, P_heterogeneity_ = <0.001). A significant reduction in the Oswestry Disability Index was also observed in women who performed a physical activity intervention compared to those who did not (z = <2.37, *p* = <0.02; SMD = −0.80, 95% CI = −1.47, −0.14, I^2^ = <91%, P_heterogeneity_ = <0.001), resulting in a significant decrease in general pain in intervention groups (z = <3.87, *p* = <0.001; SMD = −0.56, 95% CI = −0.84, −0.27, I^2^ = <86%, P_heterogeneity_ = <0.001) as shown in Figure 3. The evaluation of the publication bias of analysed articles resulted in a general absence of publication bias (*p* = <0.400). Specifically, in the first analysis (*p* = <0.580), in the second analysis (*p* = <0.826), and in the third analysis (*p* = <0.151), no publication bias in included articles was observed. 

### 3.4. Physical Activity Effects on Events of Back Pain and Pelvic Girdle Pain at the End of Pregnancy

**Figure 4:**
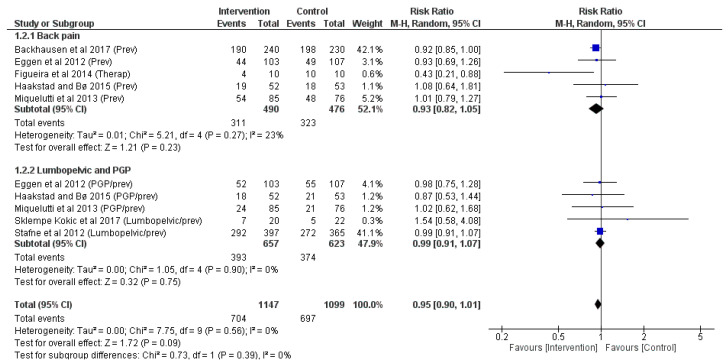
Analysis of the effects of physical activity on the presence of back pain and pelvic girdle pain events [19,21,24,25,29,32,34]. Prev (preventative effect of intervention); Therap (therapeutic effect of intervention); PGP (pelvic girdle pain).

Seven articles [19,21,24,25,29,32,34] were analysed (*n* = 1919) for events of back pain and pelvic girdle pain at the end of pregnancy per study group. No significant differences were reported between groups in back pain (z = <1.21, *p* = <0.23; RR = <0.93, 95% CI = <0.82, 1.05, I^2^ = <23%, P_heterogeneity_ = <0.27) or pelvic girdle pain (z = <0.32, *p* = <0.75; RR = <0.99, 95% CI = <0.91, 1.07, I^2^ = <0%, P_heterogeneity_ = <0.90), and generally for the presence of both types of pain (z = <1.72, *p* = <0.09; RR = <0.95, 95% CI = <0.90, 1.01, I^2^ = <0%, P_heterogeneity_ = <0.56), shown in Figure 4. After the Egger regression publication bias test, a general absence of it (*p* = <0.355) was obtained, but also in the first (*p* = <0.801) and in the second (*p* = <0.679) analyses.

## 4. Discussion

Musculoskeletal pain and discomfort are common during pregnancy and can result in poor quality of life [10,35,36]; therefore, there is research to identify effective ways to both prevent and treat this problem. The findings of this review highlight that physical activity during pregnancy may be useful in supporting women with managing symptoms of pain by reducing pain intensity and disability of pain. Although physical activity did not appear to reduce the incidence of having pain, including in various regions such as back pain or pelvic girdle pain, reducing intensity may be indicative of protecting the ability to perform activities of daily living, thereby improving quality of life throughout gestation. Importantly, although this review sought to identify any region of pain, the results retrieved predominantly focused on back and pelvic girdle pain, suggesting that further research is needed more broadly on experiences of pain and the effectiveness of physical activity during pregnancy. 

The findings of this review are similar to those of previous reviews that have found that physical activity during pregnancy can reduce the intensity and severity of pain, but may not be effective in completely preventing its occurrence [9,10,37]. Experiencing pain during pregnancy is common and can affect up to 20% to 80% of individuals and various regions of the body [2,36,38]. The etiology of pain is multifactorial and alters as pregnancy progresses caused by changing factors such as hormonal fluctuations, postural adaptations, weight gain, and fluid retention [36]. Consequently, experiencing bodily pain or discomfort may be difficult to entirely prevent, given the significant physical changes the body is going to experience with the growing foetus. For example, Bagwell et al., (2022) investigated changes in gait throughout pregnancy and compared these changes between those who had high versus low scores of low back and pelvic girdle pain [39]. Their observations highlighted that those with more pain had less contribution to total hip work and more ankle contribution, which may make them susceptible to injury or disability [39]. Therefore, effective ways to manage the symptoms of pain are essential. According to our findings and others, pregnant women experiencing pain should be recommended to engage in physical activity as a way to alleviate symptoms. Different types of exercise can be recommended depending on the location of pain / discomfort and the person’s ability, but, in general, a combination of strengthening, balance, and stretching movements should be performed [9,40,41]. In particular, physical activity and health promotion efforts should be performed with other available resources such as counselling to maintain proper spinal position (especially as the centre of gravity changes throughout pregnancy progression), support pillows and belts, and massage therapies [42,43,44,45]. 

Pain and discomfort in pregnancy may also have a direct effect on psychological outcomes as well, such as stress, poor emotional regulation, and sleep disturbances [46,47,48]. Consequently, pain and discomfort can then impact daily activities and quality of life. It is necessary to educate pregnant women about the several benefits of physical activity during pregnancy that also extend to psychological variables, such as improving overall mood, reducing fatigue, and improving sleep quality [49,50]. As medication use during pregnancy to treat pain could be avoided, physical activity can be supplemented as a noninvasive therapy option involving numerous benefits that will improve overall well-being throughout gestation [51]. In fact, a large international study that conducted a Delphi survey with pain experts aimed to understand what experts would prioritise as therapies for pregnant women experiencing pelvic girdle pain, and exercise was one of the top priorities [52].

This review further emphasises the findings of previous studies on the effectiveness of physical activity during pregnancy in reducing the intensity of physical pain and disability. Strengths of this review include screening for randomised controlled trials, inclusion of studies in not only English but also Spanish; high-quality studies being included; and a broad search that was not restricted by region of pain. Limitations include low retrieval of articles, especially evaluations in regions other than back pain or pelvic girdle pain; inability to assess performance or attrition bias, which may affect study quality; and high heterogeneity. Furthermore, it should be noted that participant pools were largely homogeneous in terms of factors such as age and body mass index, which could also influence pain throughout pregnancy. Finally, given that most of the studies included women who had pain, the ability to address prevention of pain was limited. Further research is needed to assess the effectiveness of early implementation of physical activity in pregnancy on pain prevention as pregnancy progresses, research into more body regions, the impact of supervised versus unsupervised exercise programmes on pain and disability, and other characteristics, such as pain existing before pregnancy or pain in subsequent pregnancies and the impact of physical activity. 

## 5. Conclusions

Physical activity during pregnancy was associated with a significant reduction in musculoskeletal pain intensity and disability. There was no effect of being active during pregnancy on developing back pain, pelvic girdle pain, or pain in general. These findings are in line with previous reviews that emphasise that being active during pregnancy can be a sufficient strategy to manage symptoms of pain and reduce the likelihood that pain could progressively worsen and be debilitating. Healthcare professionals should refer their pregnant patients experiencing pain to physical activity, including various types of movement, such as strengthening, stabilising, and stretching exercises.

## Figures and Tables

**Table 1:** Outcomes, interventions, and general characteristics of assessed articles.

Ref.	Country	N	IG	CG		Intervention							Main Outcomes	Secondary Outcomes	Co-Intervention
						Freq	Inten	Time	Type	Super	Dur	Adh			
Backhausen et al., 2017 [19]	Denmark	516	258	258		2	Low-Mod	12	Swimming, aerobic, and strengthening exercises in water	Unsup	45 min.	ND	Intensity of low back pain	Self-reported days spent on sick leave	-
Bolanthakodi et al., 2018 [20]	India	150	75	75		3	Mod	9	Yoga	Unsup	30 min.	ND	Labour pain intensity, type, and duration of delivery	Birth weight and preterm delivery	-
Eggen et al., 2012 [21]	Norway	257	129	128		1	ND	16–20	Aerobic, joint mobility, strengthening, and pelvic floor exercises	Sup	60 min.	ND with mean	Events of low back pain and pelvic girdle pain	Pain and disability	Home exercises
Field et al., 2012 [22]	United States	84	28	28	28	2	Mod	12	Yoga	Sup	20 min.	ND	Anxiety and depression during pregnancy	Back and legs pain	-
Field et al., 2013 [23]	United States	79	40	39		1	Mod	12	Yoga	Unsup	20 min.	ND	Anxiety and depression during and after pregnancy	Hormone levels and back and legs pain	-
Figueira et al., 2014 [24]	Brazil	40	20	20		2	Mod	11	Warm up (dancing smooth), flexibility, and relaxation	Sup	45 min.	ND	Pain intensity	Anthropometric variables	-
Haakstad and Bø, 2015 [25]	Norway	105	52	53		2	Mod	12	Aerobic, endurance, strengthening, and pelvic floor exercises	Sup	60 min.	40.4%	Low back pain and pelvic girdle pain during and after pregnancy	Pain severity	-
Kordi et al., 2013 [26]	Iran	105	35	35	35	3	Mod	ND	Aerobic, stretching, and strengthening of pelvic girdle muscles	Unsup	ND	ND	Disability due to the pain and pain intensity	Maternal characteristics and quality of life	-
Mamipour et al., 2023 [27]	Iran	35	18	17		1	ND	10	Warm-up, core stability, and cool-down exercises	Sup	60 min.	ND	Pain intensity and disability	Quality of life	-
						2				Unsup	30 min.				
Mazzarino et al., 2022 [28]	Australia	21	11	10		1	ND	6	Pilates	Sup	60 min.	72.7%	Feasibility of Pilates classes	Quality of life, pain, and mobility	Instructions and performance of daily floor work exercises at home
Miquelutti et al., 2013 [29]	Brazil	197	97	100		2	ND	16–22	Nonaerobic exercises and pelvic floor muscle training	Sup	50 min.	ND	Lumbopelvic pain, anxiety, and urinary incontinence	Perinatal outcomes	-
Mohyadin et al., 2020 [30]	Iran	84	42	42		1 every 2	ND	11	Yoga	Sup	60 min.	100%	Anxiety, labour pain, and length of labour stages	Neonatal Apgar score and mode of delivery	Booklet and training DVD to teach principles of yoga
						3				Unsup	20 min.				
Ozdemir et al., 2015 [31]	Turkey	96	48	48		3	ND	4	Stretching, tightening, and loosening movements	Sup	30 min.	ND	Change in pain intensity	Disability due to pain and frequency of low back and pelvic pain	Individualised health counselling to relieve low back and pelvic pain
									Walking exercises						
Sklempe Kokic et al., 2017 [32]	Croatia	42	22	20		2	Mod	6	Aerobic, resistance, pelvic floor, stretching, and relaxation exercises	Sup	50–55 min.	70%	Outcomes of gestational diabetes mellitus	Disability due to the pain, and pelvic girdle	-
Sonmezer et al., 2021 [33]	Turkey	40	20	20		2	ND	8	Pilates	Sup	60–70 min.	ND	Low back disability and pain intensity	Health-related quality of life and lumbopelvic stabilisation	-
Stafne et al., 2012 [34]	Norway	762	397	365		1	Mod	12	Aerobic (or endurance), strengthening, balance, stretching, and pelvic floor exercises	Sup	55–70 min.	55%	Prevalence and sick due to lumbopelvic pain	Disability due to the pain and pain intensity	-
						2				Unsup	45 min.				

Ref. (reference); N (total sample size); IG (intervention group sample size); CG (control group sample size); Freq (weekly frequency of sessions); Inten (intensity); Time (duration, in weeks, of intervention program); Super (supervision of sessions by a professional); Dur (session duration); Adh (adherence); Mod (moderate); Sup (supervised); Unsup (unsupervised); ND (not described); min. (minutes).

## Data Availability

The data presented in this study are available on request from the corresponding author.
